# A Novel Fault Diagnosis Strategy for Diaphragm Pumps Based on Signal Demodulation and PCA-ResNet

**DOI:** 10.3390/s24051578

**Published:** 2024-02-29

**Authors:** Fanguang Meng, Zhiguo Shi, Yongxing Song

**Affiliations:** 1 College of Information Science and Electronic Engineering, Zhejiang University, Hangzhou 310013, China; 2 Zhejiang JingLiFang Digital Technology Group Co., Ltd., Hangzhou 310012, China; 3 School of Thermal Engineering, Shandong Jianzhu University, Jinan 250101, China; 4 State Key Laboratory of Compressor Technology (Compressor Technology Laboratory of Anhui Province), Hefei 230031, China

**Keywords:** diaphragm pump, fault diagnosis, signal demodulation, residual network

## Abstract

The efficient and accurate identification of diaphragm pump faults is crucial for ensuring smooth system operation and reducing energy consumption. The structure of diaphragm pumps is complex and using traditional fault diagnosis strategies to extract typical fault characteristics is difficult, facing the risk of model overfitting and high diagnostic costs. In response to the shortcomings of traditional methods, this study innovatively combines signal demodulation methods with residual networks (ResNet) to propose an efficient fault diagnosis strategy for diaphragm pumps. By using a demodulation method based on principal component analysis (PCA), the vibration signal demodulation spectrum of the fault condition is obtained, the typical fault characteristics of the diaphragm pump are accurately extracted, and the sample features are enhanced, reducing the cost of fault diagnosis. Afterward, the PCA-ResNet model is applied to the fault diagnosis of diaphragm pumps. A reasonable model structure and advanced residual block design can effectively reduce the risk of model overfitting and improve the accuracy of fault diagnosis. Compared with the visual geometry group (VGG) 16, VGG19, ResNet50, and autoencoder models, the proposed model has improved accuracy by 35.89%, 80.27%, 2.72%, and 6.12%. Simultaneously, it has higher operational efficiency and lower loss rate, solving the problem of diagnostic lag in practical engineering. Finally, a model optimization strategy is proposed through model evaluation metrics and testing. The reasonable parameter range of the model is obtained, providing a reference and guarantee for further optimization of the model.

## 1. Introduction

As key equipment for industrial raw material transportation, diaphragm pumps are widely used in fields such as chemical engineering and ore processing. The internal structure of the diaphragm pump is complex. The raw materials conveyed are mostly high-temperature and highly corrosive substances, resulting in a harsh working environment [1,2,3]. During long-term operation, the one-way valve, reducer, and diaphragm of the diaphragm pump are prone to malfunctions. Timely fault diagnosis and repair are crucial for ensuring production safety [4,5]. Traditional fault diagnosis is mostly based on empirical methods, with more manpower and time costs consumed. Therefore, this article proposes a novel fault diagnosis method for diaphragm pumps based on signal demodulation and residual networks (ResNet). 

The fault diagnosis methods for diaphragm pumps are mainly divided into operating mechanism methods, operating data methods, and artificial intelligence methods [6]. Ma et al. [7] used computational fluid dynamics methods to study the operating mechanism of pumps, revealing the relationship between internal structural operating characteristics and parameter changes (operating mechanism method). Through experimental methods, simulation results were verified. Liu et al. [8] proposed an efficient fault diagnosis method based on continuous wavelet transform and least squares method for valve spring fracture faults in pumps, accurately identifying singular points of fault signals and determining spring fracture faults (operating data method). Yan et al. [9] applied the cumulative degradation estimation error method to the fault diagnosis of diaphragm pumps and proposed a diaphragm pump operation evaluation method based on the Markov model and vector autoregressive model (artificial intelligence method). The accuracy of the diaphragm pump health status evaluation was improved. Wu et al. [10] proposed a simplified numerical simulation model for the internal pressure pulsation of a diaphragm pump (operating mechanism method). The deformation and pressure pulsation characteristics of the diaphragm pump under different operating conditions were analyzed, and the accuracy of the diaphragm pump simulation was improved. Lee et al. [11] proposed an advanced estimation method for valve leakage damage in diaphragm pumps based on experimental and numerical simulation methods (operating mechanism method). The proposed mathematical model can describe the normal and valve leakage fault states of diaphragm pumps. Based on the probability characteristics of valve leakage, the probability density function of damage parameters is compared to diagnose faults. Ling et al. [12] used numerical simulation methods to analyze and improve the mechanical strength of the diaphragm chamber inside the diaphragm pump, and checked the mechanical strength (operating mechanism method). Wang et al. [13] proposed a pump fault identification method based on fuzzy logic technology (artificial intelligence method). The fuzzy information of different pump faults and operating states was analyzed, and the fuzzy relationships between different fault symptoms and events were revealed, achieving pump fault diagnosis and state recognition. Jiang et al. [14] proposed a fault diagnosis method based on symmetric polar coordinate images and clustering algorithms to address the issue of nonintuitive time-domain spectral features of pump fault signals (artificial intelligence method). The effectiveness of the diagnosis method was verified through fault classification results. The above literature has laid the foundation for the research on fault diagnosis of diaphragm pumps. However, in practical engineering, the diversity in fault types and the lag in fault diagnosis of diaphragm pumps pose challenges to traditional methods. Meanwhile, due to the difficulty in obtaining fault data and the high cost of labor and time, the existing methods make it difficult to meet the requirements of efficient fault diagnosis in practical engineering. In order to improve the diagnostic efficiency and accuracy of diaphragm pumps, this study combines signal demodulation methods with deep learning models.

Due to the working characteristics of the diaphragm pump [15,16,17], there is a modulation phenomenon in the signal spectrum. The numerous interference frequencies increase the difficulty of fault feature extraction and reduce the accuracy of fault diagnosis. The signal demodulation methods have been widely studied to obtain modulation characteristics. Antoni et al. [18] proposed the Kurtogram demodulation method based on spectral kurtosis analysis. The vibration signal is decomposed into narrowband signals through a filter, and the kurtosis coefficient is used to identify the frequency band for signal demodulation. Through the rotating machinery experiments, the effectiveness of the algorithm was verified. Moshrefzadeh et al. [19] proposed an Autogram demodulation algorithm based on spectral kurtosis analysis and the Kurtogram method. Through the Autogram method, the kurtosis of the demodulated signal is calculated. With a combined square envelope spectrum, the resonant frequency band is identified. The effectiveness of the Autogram algorithm has been verified through experimental data validation. This demodulation method based on resonance frequency band can achieve good demodulation results in high signal-to-noise ratio situations. However, when the signal-to-noise ratio of the monitoring signal decreases, its demodulation performance rapidly decreases, and its noise resistance performance is poor. In actual operation, the complex structure and background noise of the diaphragm pump result in a low signal-to-noise ratio of the signal. Traditional demodulation methods can no longer accurately obtain their modulation characteristics. Song et al. [20] proposed a signal demodulation method based on principal component analysis (DPCA). The DPCA method improves the demodulation performance of low signal-to-noise ratio signals and has high demodulation efficiency and accuracy. The effectiveness of the algorithm was verified through simulation and experimental signals. In this study, the DPCA algorithm is used to process the fault signals of diaphragm pumps, extract signal modulation characteristics, and enhance the characteristics of fault data. This lays the foundation for subsequent deep learning model fault classification, and also provides new solutions for diaphragm pump fault diagnosis problems.

To improve the efficiency of mechanical fault diagnosis, neural networks are widely used, like hydraulic pumps [21,22,23], air conditioning systems [24], and airborne fuel pumps [25]. Liu et al. [26] proposed an online fault diagnosis method based on deep transfer convolutional neural networks. The fault diagnosis method improves real-time diagnostic performance by constructing an offline cellular neural network to pre-train data. Kong et al. [27] proposed a hybrid model classification algorithm based on autoencoder (AE) for early fault detection of rotating machinery. It extracts the temporal correlation characteristics of data through acoustic emission and long-term memory networks. However, when the training set is small or the signal-to-noise ratio is reduced, such models are prone to overfitting and find difficulty in capturing complex nonlinear relationships. The comparative testing work for this type of model is carried out in Section 4.3. Zhu et al. [28] proposed an fault diagnosis model based on AlexNet to address the limitations of traditional fault diagnosis for hydraulic piston pumps. By modifying the network structure and parameters, five types of piston pump states were accurately identified. However, due to its relatively large model size and number of parameters, the computational cost of training and inference is relatively high. Secondly, the design of AlexNet can easily lead to overfitting in certain situations, especially in tasks with small amounts of data. In practical engineering, it is difficult to obtain a large amount of fault data for diaphragm pumps. Wang et al. [29] proposed a fault diagnosis method based on generative adversarial networks and stacked denoising automatic encoders. The generator and discriminator are optimized, and the sample quality and model fault recognition ability are improved. The diagnostic performance of the model is verified through reducer experiments. Wen et al. [30] proposed a deep convolutional neural network-based fault diagnosis generation adversarial learning method for the complex working conditions of the reducer. An intelligent fault diagnosis scheme was developed, and the effectiveness of the model was verified through reducer experiments. However, in the above model, the design of the network structure and the selection of hyperparameters have a significant impact on performance, requiring adjustment through experience and experimentation, resulting in high time and labor costs. In this study, in order to improve the stability of fault diagnosis models and solve the overfitting problem of traditional models, the principal component analysis (PCA) method is combined with the ResNet model to extract key features and accurately identify diaphragm pump faults using the ResNet model. At the same time, the reasonable use of signal demodulation methods enhances the characteristics of fault samples and improves the accuracy of diaphragm pump fault diagnosis.

In this study, to improve the efficiency of diaphragm pump fault diagnosis, eight vibration sensors arranged in the diaphragm pump system were used to collect three types of diaphragm pump fault signals in actual engineering. This included one-way valve failure, reducer fan failure, and diaphragm failure, and the types of faults were determined through maintenance logs. We processed raw fault data using the DPCA method, extracted signal features, and used the PCA-ResNet model to diagnose diaphragm pump faults. In Section 2, the DPCA data enhancement method and the principle of the ResNet model are introduced. In Section 3, the experimental platform and fault experimental methods are introduced. In Section 4, through comparative testing with traditional methods, the effectiveness of the DPCA method and the PCA-ResNet model is verified. In Section 5, the optimization strategy of the PCA-ResNet model is discussed, and the optimal parameter range is obtained. In Section 6, the conclusion is drawn, and the potential application of this strategy is discussed. 

The research in this article is a new attempt to combine signal demodulation methods with deep learning models. Unlike traditional strategies, the PCA method has been applied twice in this new strategy. In signal demodulation methods, PCA is used to extract principal component modulated signals to obtain stronger fault sample features. In deep learning models, the introduction of the PCA method reduces data dimensions and fault diagnosis time, which to some extent solves the problem of diagnostic lag in practical engineering. However, the computational efficiency of the model proposed in this study still needs to be improved. In future research, we will further optimize the model structure.

## 2. Research Method

**Figure 1:**
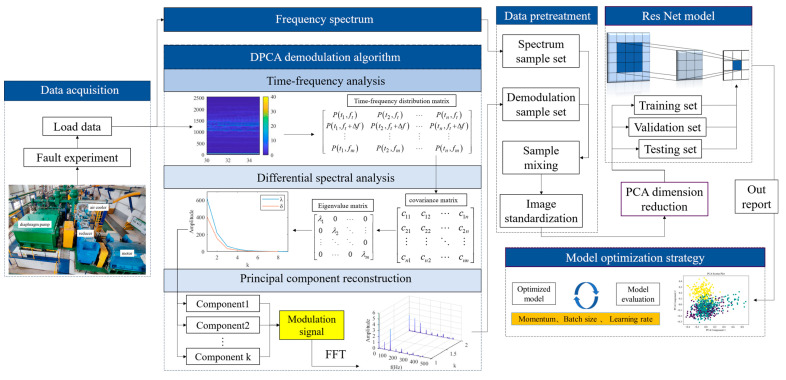
Fault diagnosis strategy of diaphragm pump based on signal demodulation and PCA-ResNet.

The structure of the diaphragm pump fault diagnosis strategy based on DPCA and PCA-ResNet is shown in Figure 1. By using the DPCA algorithm to enhance the features of fault data, the resulting enhanced images are input into the PCA-ResNet model for fault diagnosis. The fault diagnosis strategy is divided into five parts: raw data collection, data feature enhancement, data preprocessing, fault diagnosis model, and model optimization strategy. By establishing a diaphragm pump signal acquisition system and conducting long-term monitoring, three types of fault vibration signals are obtained. Then, the demodulation spectrum of the vibration signal is obtained through the DPCA method to enhance the characteristics of the fault data. To improve the stability of the model, signal demodulation spectrum preprocessing is carried out through sample mixing and image standardization. Finally, the image is input into the PCA-ResNet model, and the dimensionality of the data is reduced through the PCA method for training, validation, and testing. By evaluating the indicators of the model and conducting extensive testing, the key parameter range of the model can be obtained to improve fault diagnosis performance.

The key components of this method are the DPCA data augmentation method and the PCA-ResNet fault diagnosis model. As a signal demodulation method, the DPCA method can accurately extract the modulation characteristics of the diaphragm pump vibration signal and obtain its signal demodulation spectrum. Compared to the original data, image features are enhanced. Afterward, the obtained signal demodulation spectrum is input into the PCA-ResNet model, further extracting key features of demodulation spectrum through PCA, reducing data dimensions, and using the ResNet model for fault diagnosis. In this method, the innovative use of two rounds of PCA iteratively extracts key fault features, alleviating the problems of diagnostic lag and low success rate in practical engineering. The DPCA data augmentation method and PCA-ResNet fault diagnosis model structure will be elaborated in detail in the following text.

In this study, the algorithm code was run on a laptop with 24 GB of RAM, a 64-bit Windows 10 operating system, and an Intel Core i5-7300 processor.

### 2.1. Data Enhancement Method

**Figure 2:**
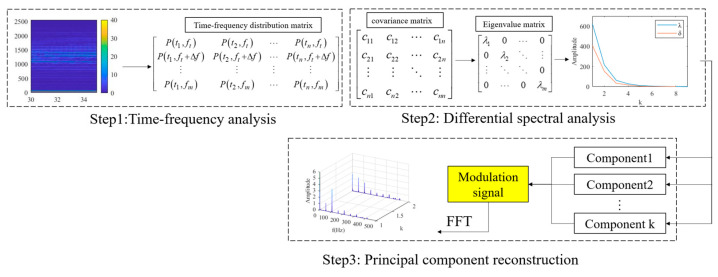
Principle of DPCA.

In this study, signal demodulation methods were applied to the field of data enhancement and the DPCA signal demodulation method was selected [31]. The DPCA method extracts modulation features of the signal. Compared with the signal spectrum, the DPCA algorithm obtains a signal demodulation spectrum with stronger fault characteristics. The principle of DPCA is shown in Figure 2. This algorithm mainly includes three parts: time–frequency analysis, differential spectrum analysis, and principal component reconstruction. Firstly, using the short-time Fourier transform method, the time–frequency distribution function of the vibration signal is obtained, and the window function is the Hanning window, as shown in Equation (1). The signal’s amplitude spectral density function is derived from the time–frequency distribution function, as shown in Equation (2). Then, the time–frequency distribution matrix is solved, as shown in Equation (3). Through the minimum limit frequency, the data dimension is changed, and the algorithm efficiency is improved.

(1) $$S_{x}(f,t)=\int_{-\infty }^{\infty }x(\tau )h(t-\tau )e^{-j\omega \tau }d\tau$$

where x(τ) is a raw signal; h(t−τ) is a window function; e is a constant; j is a unit imaginary number.

(2) $$S(f,t)=\frac{2\times \left|S_{x}(f,t)\right|}{L_{\mathrm{FFT}}}$$

where $S_{x}\left(f,t\right)$ is the time–frequency distribution function; $L_{FFT}$ is the transform length of a window function.

(3) $$S(t,f)=\left[\begin{array}{cccc} S(t_{1},f_{t}) & S(t_{2},f_{t}) & \cdots & S(t_{n},f_{t})\\ S(t_{1},f_{t}+\Delta f) & S(t_{2},f_{t}+\Delta f) & \cdots & S(t_{n},f_{t}+\Delta f)\\ \vdots & \vdots & \vdots & \vdots \\ S(t_{1},f_{m}) & S(t_{2},f_{m}) & \cdots & S(t_{n},f_{m}) \end{array}\right]$$

where $f_{t}$ is the minimum limiting frequency; $f_{m}$ is the modulated frequency.

Through the PCA method, the differential spectrum analysis is performed on the time–frequency distribution matrix, the characteristic frequency bands in the matrix are extracted, and the signal is reconstructed. In differential spectrum analysis, the first step is to calculate the covariance matrix. The covariance matrix calculation method is shown in Equation (4). Through the eigenvalue decomposition method, the eigenvalues and eigenvectors of the matrix are obtained, as shown in Equations (5)–(7). By performing differential spectral analysis on the matrix, the order of eigenvalues is selected based on the analysis results, as shown in Equation (8). The signal is rebuilt, and the principal component modulated signal is obtained by choosing the principal components. Finally, the signal demodulation spectrum is obtained through fast Fourier transform, which includes the signal modulation characteristics. Compared with the signal spectrum, the data features are enhanced and the fault diagnosis accuracy is improved.

(4) $$S_{\mathrm{cov}}=\mathrm{cov}(S(t,f))$$

where cov() is the covariance equation.

(5) $$\left[V,U]=\mathrm{eig}(S_{\mathrm{cov}}\right)$$

where V is the eigenvalue matrix and U is the eigenvector matrix.

(6) $$V=\left[\begin{array}{cccc} \lambda_{1} & 0 & \cdots & 0\\ 0 & \lambda_{2} & \ddots & \vdots \\ \vdots & \ddots & \ddots & 0\\ 0 & \cdots & 0 & \lambda_{m} \end{array}\right]$$

where $\lambda_{i}$ is the eigenvalue of the matrix.

(7) $$U=[\mu_{1},\mu_{2},\cdots ,\mu_{m}]$$

where $\mu_{i}$ is the eigenvector of the matrix.

(8) $$k\geq i{|}_{\max(\delta_{i}=(\lambda_{i}-\lambda_{i+1}))}$$

where k is the order of the characteristic value, $\delta_{i}$ is the difference value.

### 2.2. Deep Learning Model

As a typical deep learning model, convolutional neural networks (CNNs) have a wide range of applications in various fields. CNNs exhibit distinctive features such as local connectivity and weight sharing. The convolutional layer detects features in the image, such as edges, textures, and shapes, through local operations of the filter. This local connectivity reduces the number of parameters and improves network efficiency. In addition, the convolutional kernel shares weights across the entire input, reducing the complexity of the model. As shown in Figure 1, in the fault diagnosis strategy of diaphragm pumps, the signal demodulation spectrum obtained through the DPCA data enhancement method is input into the convolutional layer using its image matrix. Through matrix convolution operation, enhanced image features are extracted. The image classification and fault diagnosis are achieved through the pooling layer and the fully connected layer.

**Figure 3:**
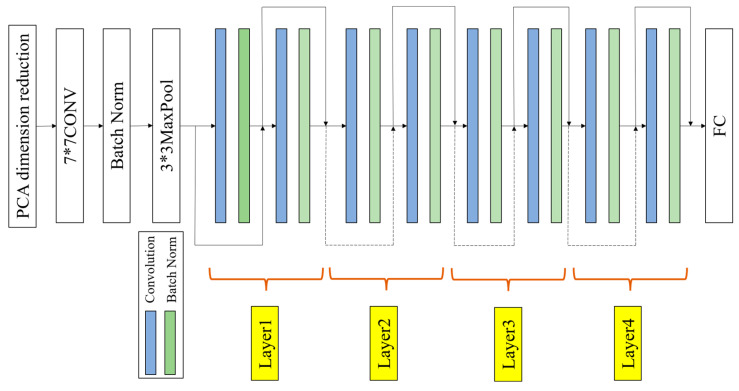
Principle of PCA-ResNet.

**Figure 4:**
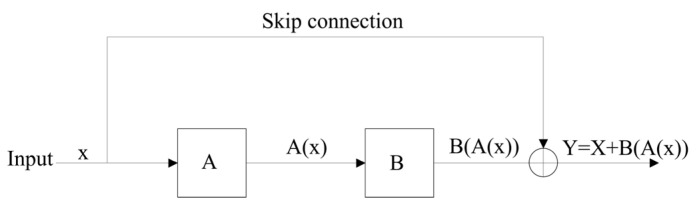
Principle of residual learning and skip connection.

As a typical CNN model, ResNet was selected in this study. In this paper, the ResNet model structure is shown in Figure 3. It includes 17 convolutional layers, 4 pooling layers, and 1 fully connected layer. The model is divided into several key stages to effectively learn and represent features. Firstly, it uses 7 × 7 convolutional kernels and 3 × 3 max pooling layers to extract preliminary features of the image. The BatchNorm layer standardizes the inputs of each batch to ensure that the inputs of each layer in the network have zero mean and unit variance. This reduces the risk of gradient vanishing and exploding and improves the training convergence speed of the network. In the following four stages, each stage has two residual blocks, and each residual block has two 3 × 3 convolutional layers. These residual blocks maintain consistency in the number of input and output channels through skip connections. Then, the feature maps are transformed into 1 × 1 format through a global average pooling layer to meet the classification requirements of the dataset. At the same time, the model introduces the concept of residual learning. The deep network training efficiency is enhanced through the design of cross-layer connections and residual blocks. The principle is to use residual connections to allow for gradients to flow more easily through the network, avoiding the problem of gradients disappearing in backpropagation, as shown in Figure 4. 

Then, to improve the diagnostic efficiency of the model and reduce the input sample dimension, the PCA method is combined with the ResNet model, and the PCA-ResNet model is proposed. In the following text, the performance of the model is discussed.

### 2.3. Evaluation Indicators for the Model

(1) Correct rate (CR)

The CR is the proportion of correctly classified samples to the total sample during training, validation, and testing. It reflects the overall performance of the fault diagnosis model. As a crucial metric for assessing model performance, the accuracy of the model in classification or prediction tasks is directly proportional to the CR. Higher CR corresponds to higher accuracy.

(2) Loss rate

The loss rate of a model is one of the key indicators for evaluating its performance. It represents the degree of difference between the predicted output of the model and the actual labels during the training process. The lower the loss rate, the better the model fits the data. When the loss rate is too low, the model will overfit, and its generalization ability will decrease. In model testing, the balance between loss rate and the generalization ability of the model is crucial.

(3) Running time

The time consumed by the PCA-ResNet model to complete a diagnosis is defined as running time. It reflects the diagnostic efficiency of the PCA-ResNet model and relates to parameters such as model maturity and data dimensions.

(4) Confusion matrix

The confusion matrix is a two-dimensional matrix and is used widely; it compares the predicted outcomes of a model with the actual observations, providing a comprehensive evaluation of their relationship. This matrix incorporates four crucial indicators: true positives (TPs), true negatives (TNs), false positives (FPs), and false negatives (FNs). The confusion matrix can be used to calculate the precision, recall, and F1 score for each type of fault.

## 3. Experimental Platform and Fault Test

### 3.1. Experimental Platform

**Figure 5:**
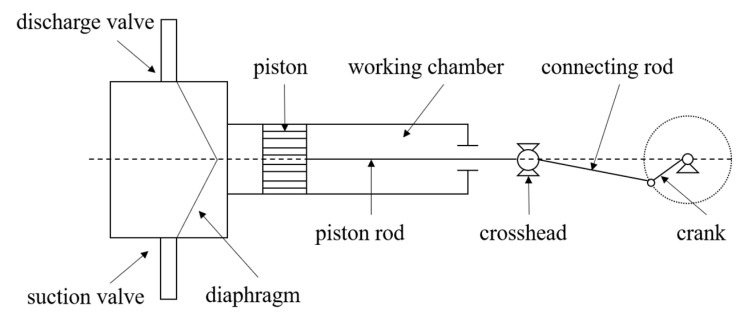
Diaphragm pump structure.

**Figure 6:**
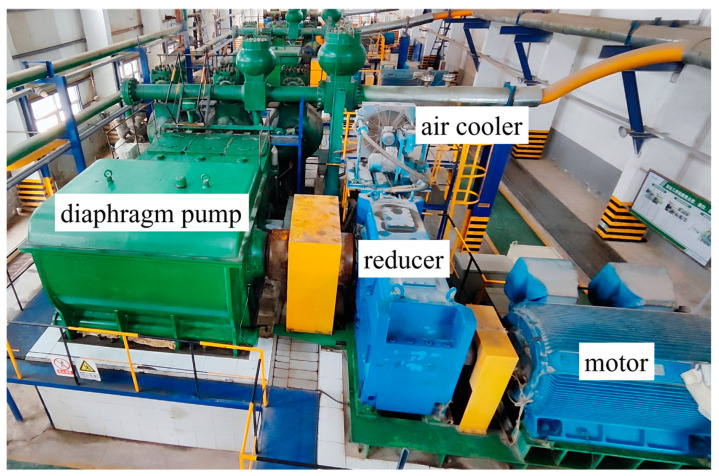
Diaphragm pump experimental platform.

The experimental platform in this study consists of a power system, a transmission system, and a multi-source signal acquisition system. The principle is shown in Figure 5 and Figure 6. The power system consists of an electric motor, a reducer, a crank–connecting rod mechanism, and a crosshead, providing power for the transmission system. The piston, diaphragm chamber, suction pipe, and discharge pipe form a transmission system for conveying raw materials. The power generated by the motor is transmitted to the crank–connecting rod mechanism and crosshead through the reducer, and the rotary operation is converted into reciprocating motion. Through the piston rod, the crosshead drives the piston to move back and forth, utilizing pressure difference changes to achieve the transportation of raw materials.

The multi-source signal acquisition system is composed of vibration acceleration sensors, laptops, and data acquisition instruments. In this study, a total of eight vibration sensors were installed on the diaphragm pump, including two in the horizontal direction of the pump body, one in the vertical direction, one on the motor drive end, one in the vertical direction of the reducer output end, two in the horizontal direction, and one in the axial direction. Through the method of multi-source signal acquisition, the operation of the power system and transmission system was scientifically evaluated, laying the foundation for the establishment of a fault diagnosis model. In this study, the experiment sampling rate is 5120 Hz.

Through a multi-source signal acquisition system, 1394 data samples of three faults were collected. In this study, all experimental data were obtained from actual engineering. The types of faults were determined through maintenance logs and provided data support for the subsequent training of neural network models. To obtain a fault diagnosis model for diaphragm pumps, the data samples were divided into three groups. Using 60% of the samples as the training set, the mapping relationship between sample features and labels was obtained by the model. Twenty percent was used as the test set to assess the model’s performance. Twenty percent was used as the validation set to verify the training effectiveness of the model and adjust hyperparameters.

### 3.2. Fault Test

In this study, the working status of the diaphragm pump was monitored through vibration sensors arranged in the system. The sensors and signal acquisition system operated continuously and stored vibration data in the server. When a malfunction occurs, the system stops running, manually checks and repairs or replaces components, and records maintenance logs to accurately classify the fault, avoiding the occurrence of false alarms. In this study, the system operates in parallel with multiple diaphragm pump units and is equipped with backup units. In the event of a single unit failure and shutdown, it will not affect the system’s operation. At the same time, to ensure uninterrupted operation of the system and minimize production costs, the following three types of faults occur in the early stages and corresponding components are directly replaced. Therefore, the degree of failure of the diaphragm pumps in this study is consistent and consistently the lowest.

(1) One-way valve fault

The one-way valve is a key component for the stable operation of a diaphragm pump. During the transmission of raw materials, irregular raw material residues can cause abnormal vibration of the valve, and corrosive raw materials can cause damage to the valve. Timely detection and repair of faults is crucial for ensuring the normal operation of diaphragm pumps. In this study, long-term multi-source signal collection was used to collect vibration signals of one-way valve faults.

(2) Reducer fan fault

An essential component of the power system, the reducer holds significant importance. The fan cools the system and dissipates heat through air cooling, which can easily cause blockage when foreign objects enter, leading to excessive temperature in the system and mechanical failure. Timely diagnosis and handling of reducer fan faults is crucial for ensuring stable power output. In this study, vibration signals of reducer fan faults were collected through long-term monitoring.

(3) Diaphragm fault

The diaphragm is the core component of a diaphragm pump. Corrosive raw materials can wear the diaphragm, thereby reducing the operating efficiency of the diaphragm pump. Therefore, when the diaphragm is worn, it should be replaced in a timely manner. This study established a logical relationship between diaphragm wear and vibration characteristics by collecting vibration signals from different directions during diaphragm pump failures.

## 4. Results and Discussion

### 4.1. Data Enhancement Results

**Figure 7:**
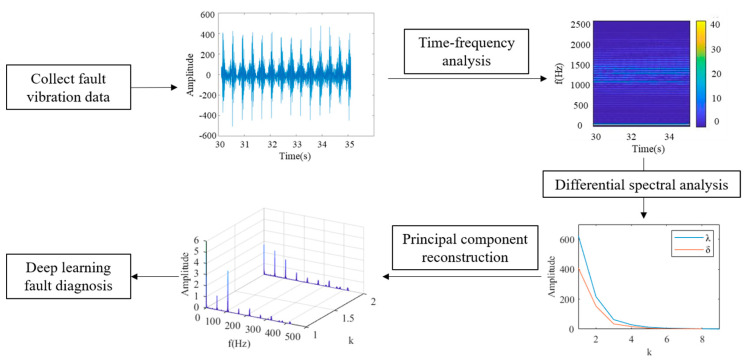
Data enhancement flow path.

**Figure 8:**
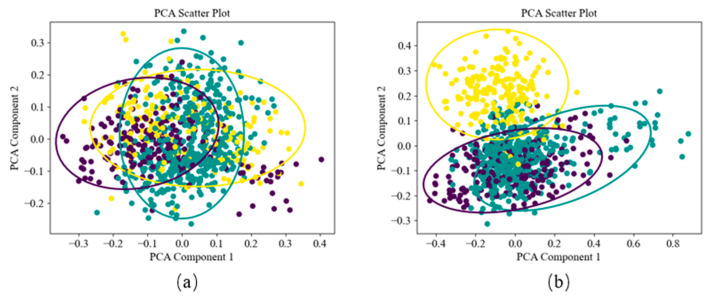
PCA results of two sample sets. (**a**) Spectrum sample set. (**b**) DPCA sample set.

In this study, DPCA was innovatively applied to the field of data enhancement. The data enhancement flow path is shown in Figure 7. Through the DPCA method, time–frequency analysis is performed on the collected fault signals, and the order of the principal components is determined through differential spectrum analysis. Then, the principal components are reconstructed to obtain the signal demodulation spectrum, which is input into the deep learning model as a fault sample. To compare and verify the effectiveness of data enhancement methods, the fast Fourier transform (FFT) method was used to obtain the spectrum of the same data as a comparison sample set. Two sample sets were input into the PCA-ResNet model, and the PCA results were obtained, as shown in Figure 8. The confidence ellipses of different colors represent a type of fault feature distribution. As shown in Figure 8a, when the spectrum is used as the sample set, the confidence ellipse coincidence rate of the three types of faults is high. Through the signal spectrum, the three types of faults cannot be effectively distinguished. As shown in Figure 8b, when the signal demodulation spectrum is used as the sample set, the distinguishability of the confidence ellipse is improved, and the data features are enhanced. This test verified the effectiveness of the DPCA data enhancement method.

**Figure 9:**
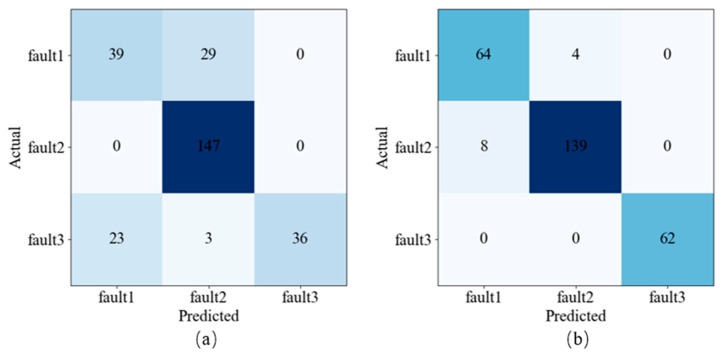
Results of the confusion matrix. (**a**) Spectrum sample set. (**b**) DPCA sample set.

The confusion matrix results for two sample sets, as shown in Figure 9 and Table 1, where fault1-3 represents one-way valve failure, reducer fan failure, and diaphragm failure. Through the data enhancement using the DPCA method, the precision of the three types of faults was 0.87, 0.97, 1.00; the recall was 0.94, 0.95, 1.00; and the F1 score was 0.91, 0.96, 1.00. Compared with the spectrum sample set, both precision and recall have improved to varying degrees, and the F1 score of the three types of faults has increased by 51.67%, 6.67%, and 36.99%. The data characteristics of the DPCA sample set are stronger, which is consistent with the results discussed earlier. The test results of the two sample sets are shown in Table 2, and the comprehensive CR, loss rate, and running time of the model are obtained. Compared with the spectrum sample set, when the model is loaded into the DPCA sample set, the CR of the model is increased by 19.38%, and the loss and running time are reduced by 77.37% and 11.22%. The comprehensive performance of the PCA-ResNet model has been improved, and this test verified the analysis above.

### 4.2. Compare and Verify with Traditional Deep Learning Model

**Figure 10:**
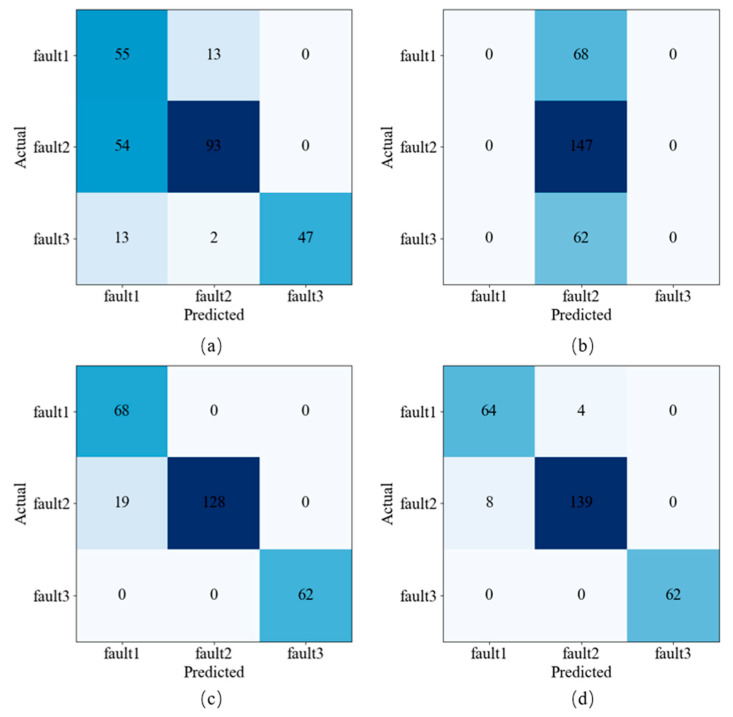
Confusing matrix results of the four models. (**a**) VGG16 (**b**) VGG19 (**c**) ResNet50 (**d**) PCA-ResNet.

In this study, the PCA-ResNet model was proposed and applied to fault diagnosis of diaphragm pumps. In this section, three other models were selected to evaluate the PCA-ResNet model through comparative testing. The VGG16, VGG19, and ResNet50 models are traditional deep learning models for image recognition. The VGG16 model consists of 13 convolutional layers, 5 pooling layers, and 3 fully connected layers, constructed using a specific architecture. The VGG19 model also has a similar design, with 16 convolutional layers, 5 pooling layers, and 3 fully connected layers. The structure of the ResNet50 model consists of 50 convolutional layers, 1 pooling layer, and 1 fully connected layer. The same model parameters are owned by four models, and the same DPCA sample set is input into four models. Figure 10 displays the results of the confusion matrix. The PCA-ResNet model has better precision, recall, and F1-score, and better fault classification and diagnosis results have been obtained for the three types of faults. The test results of four models as shown in Table 3. Compared with the other three models, when the PCA-ResNet model was applied, CR was increased by 35.89%, 80.27%, and 2.72%, Loss was reduced by 87.95%, 90.29%, and 60%, and Running time was reduced by 78.24%, 77.32%, and 59.22%. The better fault performance is owned by the PCA-ResNet model, and its effectiveness is verified.

### 4.3. Compare and Verify with Existing Fault Diagnosis Models

In this section, to further validate the performance of the proposed model, traditional model comparison verification work was carried out. The attention recurrent AE model proposed in the literature [27] was used as a comparative model. In this study, as an unsupervised algorithm, the AE model consists of two parts: encoding and decoding. The AE model is prone to overfitting training data, sensitive to input changes, and struggles to learn advanced features. In response to this deficiency, the signal demodulation algorithm is combined with the ResNet model in this article. The same fault sample set is input into two models, and the same initial parameters are set. To simplify the analysis, the three types of faults in this section are represented by OVF, RFF, and DF.

**Figure 11:**
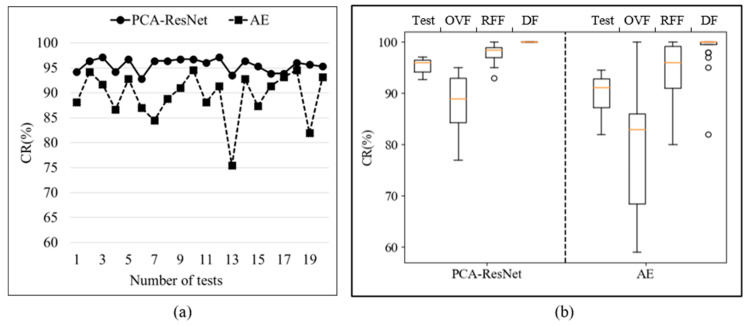
CR of 20 test results for two models. (**a**) Line chart of two models. (**b**) Box plot of two models.

A large amount of model testing work was carried out, and the CR of the two models are shown in Figure 11 and Table 4. In Figure 11a, the CR of the model test is represented. Compared with existing AE models, the model proposed in this paper has a higher fault diagnosis CR. The average CR of the AE model is 89.42%, while the average CR of the PCA-ResNet model is 95.54%, resulting in a 6.12% increase in CR. To analyze the stability of the two models, the box plots of the 20 test results are shown in Figure 11b. The main parameters of the box plot are calculated, as shown in Table 4, including Q1, Q2, and Q3, representing the lower quartile, median, and upper quartile. From the chart, the CR of OVF and RFF faults in the AE model fluctuates greatly, while there are more CR outliers of DF faults, resulting in poor model stability. Compared with the AE model, the diagnostic performance and stability of the PCA-ResNet model have been improved. The Q2 for model testing and three types of faults were increased by 4.87%, 6.00%, 2.50%, and 0%, respectively. The above results indicate that using signal demodulation methods to enhance sample features is feasible. The model proposed in this article has good diagnostic accuracy for the fault diagnosis of diaphragm pumps. Compared to traditional AE models, CR has been improved.

**Figure 12:**
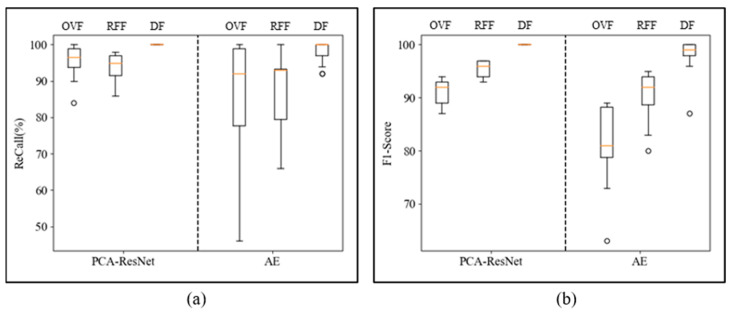
Recall and F1 score of 20 test results for two models. (**a**) Recall of two models. (**b**) F1 score of two models.

To further evaluate the diagnostic performance of each fault, the confusion matrix results of the three types of faults tested 20 times are shown in the box plot in Figure 12. Figure 12a shows the box plot of recall, and the corresponding parameters are shown in Table 5. In the test results of the AE model, there were fluctuations in the recall of all three types of faults, while the PCA-ResNet model had better recall stability and higher recall. Compared to traditional models, the Q1 of the three types of faults increased by 16%, 12%, and 3%. The F1 score box plot is shown in Figure 12b, with corresponding parameters shown in Table 6. In the test results of the AE model, there are outliers in the F1 score of the three types of faults, with Q1 and Q2 being lower. The F1 score stability of the PCA-ResNet model is better, which indirectly reflects the stability of model diagnosis. Compared to traditional models, the Q1 of the three types of faults was increased by 10.25%, 5.25%, and 2%.

**Figure 13:**
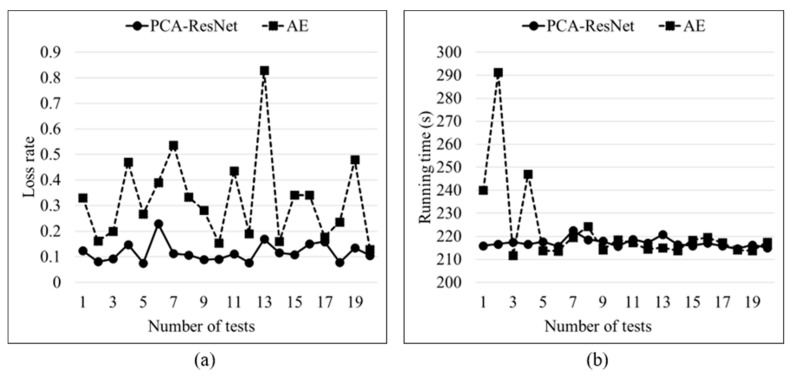
Loss rate and running time of 20 test results for two models. (**a**) Loss rate of two models. (**b**) Running time of two models.

The loss rate and running time of 20 tests for the two models are shown in Figure 13. Through comparative analysis, the loss rate of the AE model is relatively high and fluctuates greatly, while the loss rate of the PCA ResNet model is reduced and more stable. The average loss rates of the two models are 0.12% and 0.32%, and the loss rate of the PCA-ResNet model is reduced by 62.50%. The comparison of computational efficiency between the two models is shown in Figure 13b. Compared with the AE model, the proposed model in this article has more stable computational efficiency and an average running time reduced by 5.63 s.

The comprehensive test results of the two models are shown in Table 7, and the performance of the models in different aspects was evaluated through 20 tests. Compared with the AE model, the PCA-ResNet model proposed in this article improved the CR by 6.12%, further enhancing the accuracy of diaphragm pump fault diagnosis. The loss rate decreased by 62.50%, and the similarity between the model’s predicted results and the actual fault results increased. The running time was reduced by 5.63 s, and the model’s computation was improved through data augmentation methods and a reasonable network structure, reducing the time cost of fault diagnosis.

In summary, when diagnosing diaphragm pump faults, the PCA-ResNet model proposed in this paper is more stable, with higher diagnostic accuracy and computational efficiency. The DPCA algorithm can effectively enhance sample features, and the residual structure in the ResNet model improves diagnostic stability and accuracy. The effectiveness and development potential of combining signal demodulation algorithms with ResNet models have been verified.

## 5. PCA-ResNet Model Optimization Strategy

In this section, an optimization strategy based on the PCA-ResNet model is proposed. The reference range of key parameters of the model is determined.

### 5.1. Test Results of Different Batch Sizes

**Figure 14:**
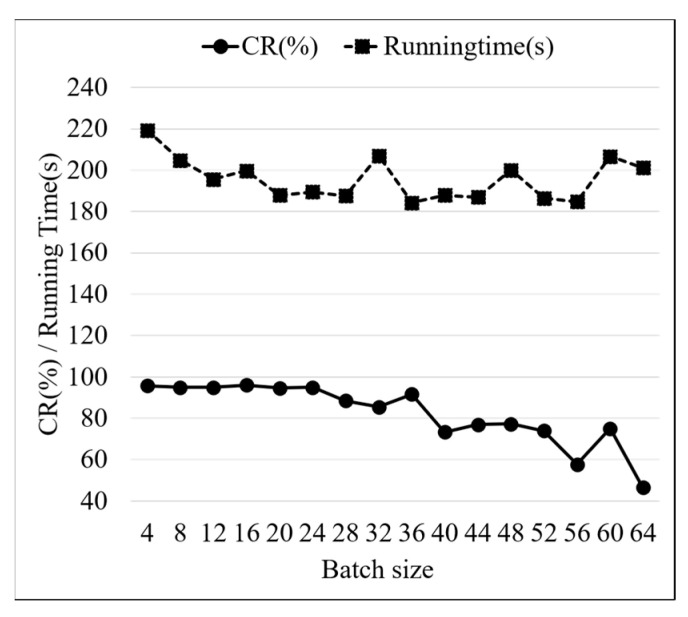
PCA-ResNet model test results of different batch sizes.

Batch size is an important parameter in deep learning models, and it determines the number of samples used to update parameters in each model training iteration. When the batch size is large, higher iteration speed and training efficiency are obtained by the model, and more memory and computing resources are consumed. When the batch size is small, the convergence speed of the model is improved, and the generalization ability is reduced. The model test results for different batch sizes are shown in Figure 14. When the batch size is greater than 24, the CR fluctuation decreases, the model is underfitted, and the feature extraction of the training data by the model is insufficient. The reasonable batch size range is 16–24, within which higher CR and lower running time are obtained, and the fault diagnosis performance of the model is improved.

### 5.2. Test Results of Different Momentum

**Figure 15:**
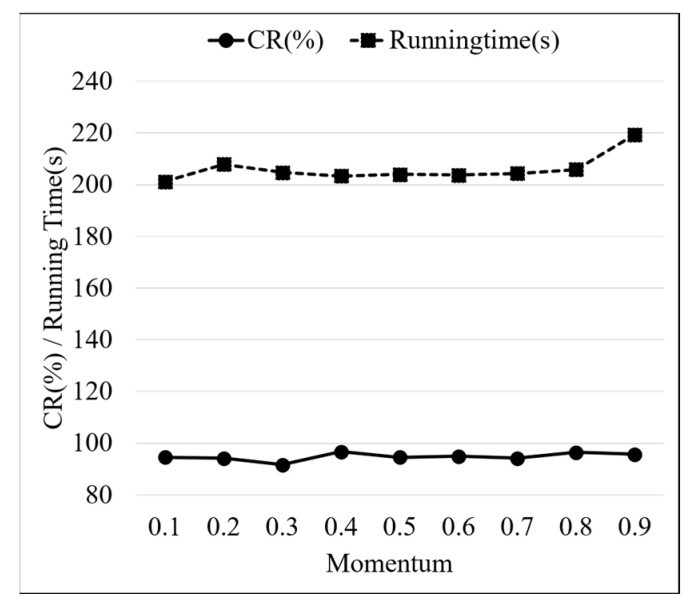
PCA-ResNet model test results of different momentums.

As the core parameter of the gradient descent optimization algorithm, momentum represents the weight of historical gradients in the current update. By evaluating the influence weights of historical gradients, the direction and speed of parameter updates are adjusted. It helps to improve the speed of updates when gradients have consistent directions in continuous updates. When the momentum is high, it may cause the model to oscillate or miss the best advantage. Figure 15 displays the test results of different momentum models. With the momentum increase, the CR and running time of the model are slowly increased. For the fault diagnosis of diaphragm pumps with small sample sizes, the reasonable momentum range is 0.4–0.8. In this range, a better fault diagnosis performance is achieved by the PCA-ResNet model.

### 5.3. Test Results of Different Learning Rates

**Figure 16:**
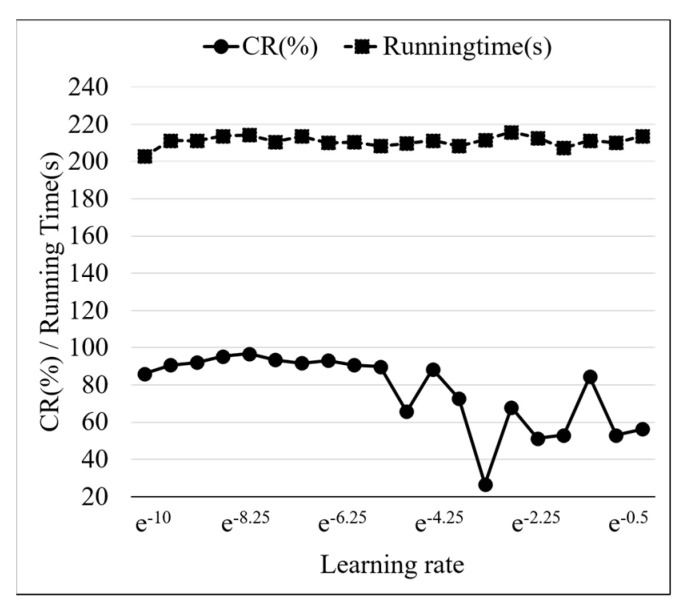
PCA-ResNet model test results of different learning rates.

Learning rate is an important hyperparameter in neural network training; it represents the step size of weight updates. When the learning rate is high, a higher convergence speed of the model is obtained, but the risk of local oscillation and non-convergence is increased. When the learning rate is low, better model stability and generalization ability are obtained, but the convergence speed is reduced, and the selection of the learning rate is crucial. Figure 16 shows the test results of different learning rate models, with the learning rate set to exponential growth. When it is less than exp (−5.25), CR and running time are stable and maintained at a high level. When it is greater than exp (−5.25), the model experiences local oscillation and cannot converge, with a minimum CR value of 26.71%. Therefore, the reasonable learning rate range is exp (−10) to exp (−5.25).

## 6. Conclusions

In this study, a fault diagnosis strategy for diaphragm pumps based on signal demodulation and PCA-ResNet was proposed. The fault signal is collected through the multi-source signal acquisition system. Through the DPCA data enhancement method, the signal demodulation spectrum is obtained and input into the PCA-ResNet model. The model optimization strategy is proposed through model testing and evaluation metrics. The following are the primary conclusions:

(1) The DPCA data enhancement method can effectively enhance data features. Compared with the spectrum sample set, the CR of the model was increased by 19.38%, and the loss rate and running time were reduced by 77.37% and 11.22%.
(2) PCA-ResNet can diagnose diaphragm pump faults efficiently. Compared with VGG16, VGG19, and ResNet50 models, CR was increased by 35.89%, 80.27%, and 2.72%, the loss rate was reduced by 87.95%, 90.29%, and 60%, and running time was reduced by 78.24%, 77.32%, and 59.22%.
(3) Compared with existing diagnostic models, the PCA-ResNet model has better stability and higher diagnostic CR. Compared to the AE model, the average CR was increased by 6.12%, the average loss rate was reduced by 62.5%, and the average running time was reduced by 5.63 s.
(4) In the PCA-ResNet model with a small sample, the reasonable batch size range is 16–24, the momentum range is 0.4–0.8, and the reasonable learning rate range spans from exp(−10) to exp(−5.25). In this range, better fault diagnosis performance is achieved by the PCA-ResNet model.

## Figures and Tables

**Table 1:** Confusion matrix results for three types of faults.

	Parameter	Precision	Recall	F1 Score	Support
Fault Sample					
Fault1 (FFT)		0.63	0.57	0.60	68
Fault2 (FFT)		0.82	1.00	0.90	147
Fault3 (FFT)		1.00	0.58	0.73	62
Fault1 (DPCA)		0.89	0.94	0.91	68
Fault2 (DPCA)		0.97	0.95	0.96	147
Fault3 (DPCA)		1.00	1.00	1.00	62

**Table 2:** Model test results of two sample sets.

	Parameter	CR (%)	Loss	Running Time (s)
Sample Set				
Spectrum		80.14	0.44	247.06
DPCA		95.67	0.10	219.36

**Table 3:** Test Results of the four models.

	Parameter	CR (%)	Loss	Running Time (s)
Model				
VGG16		70.40	0.83	1008.14
VGG19		53.07	1.03	967.23
ResNet50		93.14	0.25	537.89
PCA-ResNet		95.67	0.10	219.36

**Table 4:** Parameters of CR box plot.

	Parameter	Q1 (%)	Q2 (%)	Q3 (%)
Box				
PCA-ResNet	Test	94.22	96.03	96.48
	OVF	84.25	89.00	93.00
	RFF	97.00	98.50	99.00
	DF	100.00	100.00	100.00
AE	Test	87.27	91.16	92.87
	OVF	68.50	83.00	86.00
	RFF	91.00	96.00	99.25
	DF	99.50	100.00	100.00

**Table 5:** Parameters of recall box plot.

	Parameter	Q1 (%)	Q2 (%)	Q3 (%)
Box				
PCA-ResNet	OVF	93.75	96.50	99.00
	RFF	91.50	95.00	97.00
	DF	100.00	100.00	100.00
AE	OVF	77.75	92.00	99.00
	RFF	79.50	93.00	93.25
	DF	97.00	100.00	100.00

**Table 6:** Parameters of F1 score box plot.

	Parameter	Q1 (%)	Q2 (%)	Q3 (%)
Box				
PCA-ResNet	OVF	89.00	92.00	93.00
	RFF	94.00	96.00	97.00
	DF	100.00	100.00	100.00
AE	OVF	78.75	81.00	88.25
	RFF	88.75	92.00	94.00
	DF	98.00	99.00	100.00

**Table 7:** Comprehensive test results of the two models.

	Parameter	CR (%)	Loss	Running Time (s)
Model				
AE		89.42	0.32	222.76
PCA-ResNet		95.54	0.12	217.13

## Data Availability

The data presented in this study are available on request from the corresponding author.
